# Supplementary figures and images for: Diversification and historical demography of Haloxylon ammodendron in relation to Pleistocene climatic oscillations in northwestern China
Source: PeerJ. 2022 Dec 13;10:e14476. doi: 10.7717/peerj.14476 (PMC9756866; doi:10.7717/peerj.14476)

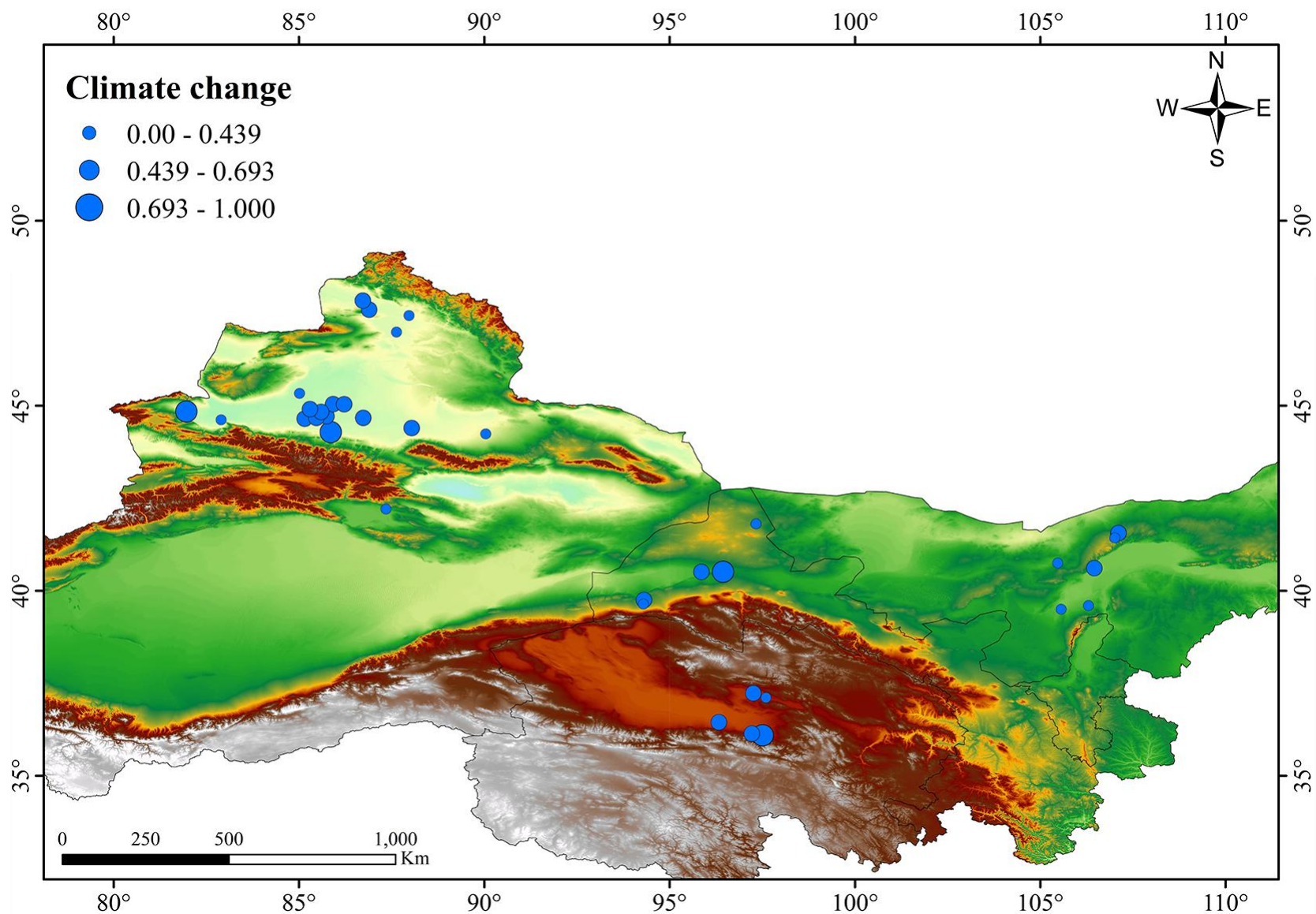

Supplement: Supplemental Information 1 — The sizes of black dots represent the change of climate variable since the LGM. The colors in figure from blue to red represent the possibility of species potential dispersal from low to high. [file peerj-10-14476-s001.pdf]
