# Supplementary material for: Diversification and historical demography of Haloxylon ammodendron in relation to Pleistocene climatic oscillations in northwestern China
Source: PeerJ. 2022 Dec 13;10:e14476. doi: 10.7717/peerj.14476 (PMC9756866; doi:10.7717/peerj.14476)
Supplement: Supplemental Information 3 — Numbers within populations indicate the number of individuals with that ribotype. Private ribotypes in each population, and populations that harboured private haplotypes, are shown in bold. [file peerj-10-14476-s003.docx]

Table S2 Distribution of 16 nDNA ribotypes in populations of *Haloxylon ammodendron*. Numbers from 1 to 16 correspond to each of the 16 ribotypes labelled in Fig. 1. Numbers within populations indicate the number of individuals with that ribotype. Private ribotypes in each population, and populations that harboured private haplotypes, are shown in bold

|  | R1 | R2 | R3 | R4 | R5 | R6 | R7 | **R8** | R9 | R10 | R11 | R12 | R13 | R14 | R15 | R16 |
| --- | --- | --- | --- | --- | --- | --- | --- | --- | --- | --- | --- | --- | --- | --- | --- | --- |
| XBL | 11 | 1 |  |  |  |  |  |  |  |  |  |  |  |  |  |  |
| XGH | 5 | 1 | 4 | 3 |  |  |  |  |  |  |  |  |  |  |  |  |
| XKM | 12 |  |  |  |  |  |  |  |  |  |  |  |  |  |  |  |
| XST | 12 | 2 |  |  |  |  |  |  |  |  |  |  |  |  |  |  |
| XSB | 5 | 11 |  |  |  |  |  |  |  |  |  |  |  |  |  |  |
| XSE |  |  |  |  | 13 |  |  |  |  |  |  |  |  |  |  |  |
| XBT |  |  |  |  |  | 5 | 6 |  |  |  |  |  |  |  |  |  |
| XBB |  |  |  |  |  | 3 | 7 |  |  |  |  |  |  |  |  |  |
| XWG | |  |  |  |  | 1 | 14 |  |  |  |  |  |  |  |  |  |
| XSF |  |  |  |  |  | 5 | 6 |  |  |  |  |  |  |  |  |  |
| XFH |  |  |  |  |  | 4 | 8 |  |  |  |  |  |  |  |  |  |
| XQT |  |  |  |  |  | 1 | 7 |  |  |  |  |  |  |  |  |  |
| XFK |  |  |  |  |  | 2 | 5 |  |  |  |  |  |  |  |  |  |
| XSG |  |  |  |  |  | 2 | 6 |  |  |  |  |  |  |  |  |  |
| **XHS** |  |  |  |  |  |  |  | 11 |  |  |  |  |  |  |  |  |
| XSW |  | 1 | 2 | 1 | 4 |  |  |  |  |  |  |  |  |  |  |  |
| XSE |  |  |  |  | 13 |  |  |  |  |  |  |  |  |  |  |  |
| XSC | 10 | 5 |  |  |  |  |  |  |  |  |  |  |  |  |  |  |
| XSD |  |  | 7 | 7 | 1 |  |  |  |  |  |  |  |  |  |  |  |
| GHM | |  |  |  |  |  |  |  | 5 | 6 | 1 |  |  |  |  |  |
| GNT |  |  |  |  |  |  |  |  | 5 | 3 | 2 |  |  |  |  |  |
| GGB |  |  |  |  |  |  |  |  | 11 |  |  |  |  |  |  |  |
| GMZ |  |  |  |  |  |  |  |  | 2 | 5 | 4 |  |  |  |  |  |
| MZQ | |  |  |  |  |  |  |  |  | 5 | 5 |  |  |  |  |  |
| MWL | |  |  |  |  |  |  |  | 3 |  | 1 | 5 | 3 |  |  |  |
| MDK | |  |  |  |  |  |  |  | 5 | 4 | 2 | 1 | 1 |  |  |  |
| MWS | |  |  |  |  |  |  |  | 3 | 4 | 2 | 2 |  |  |  |  |
| MWH | |  |  |  |  |  |  |  | 4 | 3 | 2 | 3 |  |  |  |  |
| MJL |  |  |  |  |  |  |  |  | 3 | 4 | 2 | 1 |  |  |  |  |
| QDL |  |  |  |  |  |  |  |  |  |  |  |  |  | 8 | 8 |  |
| QTS |  |  |  |  |  |  |  |  |  |  |  |  |  | 7 | 1 | 2 |
| QZJ |  |  |  |  |  |  |  |  |  |  |  |  |  | 11 |  |  |
| QGZ |  |  |  |  |  |  |  |  |  |  |  |  |  | 12 |  |  |
| QBL |  |  |  |  |  |  |  |  |  |  |  |  |  | 11 |  |  |
| QTL |  |  |  |  |  |  |  |  |  |  |  |  |  |  | 10 | 1 |
| QGH |  |  |  |  |  |  |  |  |  |  |  |  |  | 12 |  |  |
