# Supplementary material for: Diversification and historical demography of Haloxylon ammodendron in relation to Pleistocene climatic oscillations in northwestern China
Source: PeerJ. 2022 Dec 13;10:e14476. doi: 10.7717/peerj.14476 (PMC9756866; doi:10.7717/peerj.14476)
Supplement: Supplemental Information 5 — The first two variables (and the values) that have the most high contributions to the first two principal components, are shown in bold. [file peerj-10-14476-s005.docx]

Table S4 Contributions of variation range of 6 climate variables and 4 soil variables of 36 *Haloxylon ammodendron* populations in the present in Principal component analysis (PCA). The first two variables (and the values) that have the most high contributions to the first two principal components, are shown in bold

|  | PC1 | PC2 | PC3 | PC4 | PC5 | PC6 | PC7 | PC8 | PC9 | PC10 |
| --- | --- | --- | --- | --- | --- | --- | --- | --- | --- | --- |
| Bio2 | 0.321 | 0.298 | 0.181 | 0.388 | 0.13 | 0.375 | 0.632 | 0.26 | NA | NA |
| **Bio4** | **-0.377** | 0.208 | NA | -0.285 | 0.154 | -0.123 | 0.343 | NA | -0.278 | -0.706 |
| **Bio6** | **0.377** | -0.181 | NA | NA | NA | 0.571 | -0.347 | -0.277 | NA | -0.539 |
| **Bio13** | NA | **-0.659** | 0.398 | -0.309 | 0.436 | NA | 0.194 | 0.145 | 0.244 | NA |
| **Bio14** | **-0.379** | NA | 0.238 | 0.102 | NA | 0.348 | -0.341 | 0.545 | -0.484 | 0.124 |
| **Bio15** | **0.387** | -0.192 | NA | -0.115 | 0.116 | -0.178 | 0.173 | -0.226 | -0.783 | 0.229 |
| **DEM** | **0.356** | -0.171 | 0.12 | 0.316 | -0.269 | -0.54 | -0.126 | 0.459 | NA | -0.372 |
| pH | 0.281 | 0.169 | 0.148 | -0.696 | -0.534 | 0.181 | NA | 0.248 | NA | NA |
| SM | -0.289 | -0.243 | 0.515 | 0.234 | -0.551 | NA | 0.24 | -0.408 | NA | NA |
| **SC** | -0.165 | **-0.493** | -0.664 | NA | -0.306 | 0.203 | 0.32 | 0.218 | NA | NA |
