# Supplementary material for: Diversification and historical demography of Haloxylon ammodendron in relation to Pleistocene climatic oscillations in northwestern China
Source: PeerJ. 2022 Dec 13;10:e14476. doi: 10.7717/peerj.14476 (PMC9756866; doi:10.7717/peerj.14476)
Supplement: Supplemental Information 6 — ■, GTTA; , ACAA; ∘, TGG; , CC; , AT; * , CAC; △, ACA. ‘–’ represents indels with a length equal to 1 bp [file peerj-10-14476-s006.docx]

Table S5 Variable nucleotide sites for ITS regions in 14 ribotypes of *Haloxylon ammodendron*

■, GTTA; ▲, ACAA; ●, TGG; ▼, CC; ◆, AT; ★, CAC; △, ACA. ‘–’ represents indels with a length equal to 1 bp

| Ribotypes | ITS1-ITS4 | | | | | | | | | | | | | | | | | | | | | | | |
| --- | --- | --- | --- | --- | --- | --- | --- | --- | --- | --- | --- | --- | --- | --- | --- | --- | --- | --- | --- | --- | --- | --- | --- | --- |
|  | 19 | 27 | 52 | 53 | 1  2  1 | 168 | 173 | 185 | 323 | 355 | 360 | 390 | 404 | 420 | 449 | 479 | 480 | 481 | 490 | 502 | 524 | 525 | 529 | 5  3  8 |
| R1 | – | – | T | – | – | – | ▼ | – | G | – | C | A | - | A | G | G | C | A | C | G | G | ★ | – | – |
| R2 | – | – | T | – | – | – | ▼ | – | G | – | C | G | - | A | G | G | C | A | C | G | G | ★ | – | – |
| R3 | – | – | T | – | – | – | ▼ | – | G | – | C | A | - | A | G | G | - | A | C | G | - | ★ | – | – |
| R4 | – | – | T | – | – | – | ▼ | – | G | – | C | G | - | A | G | G | - | A | C | G | - | ★ | – | – |
| R5 | – | – | T | – | T | – | ▼ | T | – | – | C | G | - | A | G | G | C | - | C | G | G | ★ | – | – |
| R6 | – | – | T | – | T | – | ▼ | T | G | – | C | G | - | A | G | G | C | - | C | G | G | ★ | – | – |
| R7 | – | – | T | – | T | – | ▼ | T | G | – | C | A | - | A | G | G | C | - | C | G | G | ★ | – | – |
| R8 | – | – | T | – | T | – | ▼ | T | G | – | T | G | - | G | G | G | C | - | C | G | G | ★ | – | – |
| R9 | ■ | ▲ | A | ● | T | ▼ | ◆ | T | G | T | C | G | G | G | G | G | C | A | C | G | G | △ | C | C |
| R10 | ■ | ▲ | A | ● | T | ▼ | ◆ | T | G | T | C | A | G | A | G | G | C | A | C | G | G | △ | C | C |
| R11 | ■ | ▲ | A | ● | T | ▼ | ◆ | T | G | T | C | A | A | A | A | G | C | A | C | G | G | △ | C | C |
| R12 | ■ | ▲ | A | ● | T | ▼ | ◆ | T | G | T | C | A | A | A | G | G | C | A | C | G | G | △ | C | C |
| R13 | ■ | ▲ | A | ● | T | ▼ | ◆ | T | G | T | C | A | A | G |  | A | C | A | T | G | G | △ | C | C |
| R14 | ■ | ▲ | A | ● | T | ▼ | ◆ | T | G | T | C | A | A | A |  | G | C | A | C | G | G | △ | C | C |
