# Supplementary material for: Diversification and historical demography of Haloxylon ammodendron in relation to Pleistocene climatic oscillations in northwestern China
Source: PeerJ. 2022 Dec 13;10:e14476. doi: 10.7717/peerj.14476 (PMC9756866; doi:10.7717/peerj.14476)
Supplement: Supplemental Information 7 — ■, TTTTTTTATGAATCCG; , TCCGTTTTTTTTATGAA; ∘, GATTTGACTT; , TGACT; , TGAC; * , CT; △, AATA. ‘–’ represents indels with a length equal to 1 bp [file peerj-10-14476-s007.docx]

Table S6 Variable nucleotide sites for two chloroplast DNA regions in 24 haplotypes of *Haloxylon ammodendron*

■, TTTTTTTATGAATCCG; ▲, TCCGTTTTTTTTATGAA; ●, GATTTGACTT; ▼, TGACT; ◆, TGAC; ★, CT; △, AATA. ‘–’ represents indels with a length equal to 1 bp

| Haplotypes |  |  |  |  |  |  | *trn*S-*trn*G | | | | | | | | | | | | | | | | *trn*V | | | | | | | | |
| --- | --- | --- | --- | --- | --- | --- | --- | --- | --- | --- | --- | --- | --- | --- | --- | --- | --- | --- | --- | --- | --- | --- | --- | --- | --- | --- | --- | --- | --- | --- | --- |
|  | 2 | 3 | 6 | 8 | 1  0 | 1  9 | 2  0 | 1  1  4 | 1  5  1 | 3  5  8 | 3  5  9 | 3  7  7 | 3  9  0 | 4  9  1 | 4  9  2 | 5  0  2 | 6  7  6 | 7  7  7 | 7  9  2 | 7  9  4 | 8  1  6 | 8  2  8 | 2 | 6  7 | 8  7 | 1  1  9 | 2  2  4 | 3  2  8 | 3  3  8 | 3  7  0 | 4  7  4 |
| H1 | T | A | G | A | G | T | C | T | T | C | – | T | ▲ | - | G | C | G | A | A | C | T | T | ● | ◆ | ★ | C | G | G | G | △ | C |
| H2 | T | A | G | A | G | T | C | T | T | C | ■ | T | – | - | G | C | G | A | A | C | T | T | ● | ◆ | ★ | C | G | G | G | △ | C |
| H3 | T | A | G | A | G | T | C | T | T | C | – | T | ▲ | - | G | C | G | A | A | C | T | T | ● | ◆ | ★ | C | G | G | G | △ | C |
| H4 | T | A | G | A | G | T | C | T | T | C | – | T | ▲ | - | G | C | G | A | A | C | T | T | ● | ◆ | ★ | C | G | G | G | △ | C |
| H5 | T | A | G | A | G | T | C | T | - | C | – | T | ▲ | G | - | C | G | A | A | C | T | T | ● | ◆ | ★ | C | G | G | G | △ | T |
| H6 | T | A | G | A | G | T | C | T | - | C | – | T | ▲ | G | - | C | G | A | A | C | T | T | ● | ◆ | ★ | C | G | G | G | △ | C |
| H7 | T | A | G | A | G | T | C | T | - | C | – | T | ▲ | G | - | C | G | A | A | C | T | T | ● | ◆ | ★ | C | G | G | G | △ | C |
| H8 | T | A | G | A | G | T | C | T | T | C | – | T | ▲ | - | G | C | G | A | A | C | T | T | ● | ◆ | ★ | A | G | G | G | △ | C |
| H9 | T | A | G | A | G | T | C | T | T | - | – | C | – | - | G | C | G | A | T | C | T | T | ● | ◆ | – | C | G | G | G | △ | C |
| H10 | T | A | G | A | G | T | C | T | T | C | – | T | ▲ | - | G | C | G | A | A | C | T | T | ● | ◆ | ★ | C | G | G | G | △ | T |
| H11 | T | A | G | A | G | T | C | T | T | C | – | T | – | - | G | C | G | A | A | C | T | T | ● | ◆ | ★ | C | G | G | G | △ | C |
| H12 | T | A | G | A | G | T | C | T | - | C | – | T | ▲ | G | - | C | G | A | A | C | T | T | ● | ◆ | – | C | G | G | G | △ | C |
| H13 | T | A | G | A | G | T | C | T | - | C | – | T | – | G | - | C | G | A | A | C | T | T | ● | ◆ | ★ | C | G | G | G | △ | C |
| H14 | G | T | A | - | A | T | C | T | - | C | – | T | ▲ | G | - | T | C | T | A | T | T | A | A | – | – | ★ | C | G | T | G | – |
| H15 | G | T | A | - | A | T | C | T | - | C | – | T | ▲ | G | - | T | C | T | A | T | T | A | A | – | – | ★ | C | G | T | G | – |
| H16 | G | T | A | - | A | T | C | T | - | C | – | T | ▲ | G | - | T | C | T | A | T | T | A | A | – | – | ★ | C | G | T | G | – |
| H17 | G | T | A | - | A | T | C | C | - | C | – | T | ▲ | G | - | T | C | T | A | T | T | A | A | – | – | ★ | C | G | T | G | – |
| H18 | G | T | A | - | A | - | T | C | - | C | – | T | ▲ | G | - | T | C | T | A | T | T | A | A | – | – | ★ | C | G | T | G | – |
| H19 | G | T | A | - | A | - | T | C | - | C | – | T | ▲ | G | - | T | C | T | A | T | - | A | A | – | – | ★ | C | G | T | G | – |
| H20 | G | T | A | - | A | T | C | T | - | C | – | T | ▲ | G | - | T | C | T | A | T | - | A | A | – | – | ★ | C | G | T | G | – |
| H21 | G | T | A | - | A | T | C | T | - | C | – | T | ▲ | G | - | T | C | T | A | T | - | A | A | – | – | ★ | C | G | T | G | – |
| H22 | G | T | A | - | A | T | T | C | - | C | – | T | ▲ | G | - | T | C | T | A | T | T | A | A | – | – | ★ | C | A | T | G | – |
| H23 | G | T | A | - | A | T | C | C | - | C | – | T | ▲ | G | - | T | C | T | A | T | T | A | A | – | – | ★ | C | G | T | G | – |
| H24 | G | T | A | - | A | T | C | C | - | C | – | T | ▲ | G | - | T | C | T | A | T | T | A | A | – | – | ★ | C | G | T | C | – |
